# Three different situations and approaches in the management for anomalous origin of the right coronary artery from the left coronary sinus: case report

**DOI:** 10.1186/1749-8090-9-21

**Published:** 2014-01-23

**Authors:** Woon Heo, Ho-Ki Min, Do Kyun Kang, Hee Jae Jun, Youn-Ho Hwang, Hyung Chae Lee

**Affiliations:** 1 Department of Thoracic and Cardiovascular Surgery, Haeundae Paik Hospital, Inje University College of Medicine, 875 (Jwadong) Haeundae-ro, Haeundaegu, Busan 612-030, Korea; 2 Department of Thoracic and Cardiovascular Surgery, National Masan Tuberculosis Hospital, Busan, Korea

**Keywords:** *Coronary artery anomaly*, *Anomaly*

## Abstract

Anomalous origin of the right coronary artery from the left coronary sinus is rare but potentially dangerous if any ischemic signs are present. Multiple therapeutic options were advocated so far. We experienced three different situations and surgical approaches to these anomalies, and reviewed retrospectively. For the first case, we made a neo-ostium on the right sinus of Valsalva and anastomosed with the right coronary artery after arteriotomy. For the second and third cases, we applied coronary artery bypasses emergently: patient 2 the gastroepiploic artery during off-pump coronary artery bypass and patient 3 the left internal thoracic artery during surgery for acute aortic dissection. For the better outcomes, it is important to understand anatomic and hemodynamic characteristics of each patient and select the surgical options considering each characteristic.

## Background

Anomalous origin of the right coronary artery from the left sinus of Valsalva (ARCA) is a rare congenital anomaly but a common cause of sudden death in the young [1]. It is commonly identified incidentally by angiography, during cardiac operation, or at autopsy [2]. The fact that these patients are usually asymptomatic but could initially present with sudden death can make their management challenging. We report 3 successful surgical outcomes in patients with ARCA in different situations and approaches.

## Case presentation

Three Koreans with ARCA who had different characteristics were performed operations in our institute, and the medical records were reviewed retrospectively. The characteristics and operative procedures were described in Table 1. Two cases (patient 1 and patient 2) were diagnosed preoperatively and one case (patient 3) was identified incidentally during surgery.

**Table 1:** Summaries of characteristics of patients and operative procedures

**Sex/age**	**Operative timing**	**Type of procedure**	**Concomitant disease**	**Associated procedures**	**Result**	**F/U (months)**	**Course**
F / 39	Elective	Neo-ostium formation	None	None	Alive	25	EM
F / 61	Emergency	OPCAB using RGEA	AMI with 3VDs	OPCAB using ITAs	Alive	17	EM
M/ 44	Emergency	CABG using LITA	AAD	AA & PA replacement	Alive	9	IM

F/U; follow-up, AAD; acute type A aortic dissection, AMI with 3VDs; acute myocardial infarction with triple vessel disease, OPCAB; Off-pump coronary artery bypass, ITAs; internal thoracic arteries, CABG; coronary artery bypass graft, RGEA; right gastroepiploic artery, LITA; left internal thoracic artery; EM; extra-mural course, IM; intramural course, AA & PA replacement: ascending aorta and partial arch replacement.

### Patient 1>

**Figure 1:**
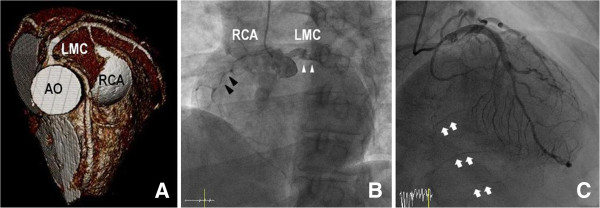
**Pre-operative images. (A)** Three dimensional volume rendering of contrast-enhanced CT scan shows the anomalous RCA shared the same sinus with the left coronary system, with its attenuated proximal portion passing between the aorta and pulmonary trunk in patient 1. The left coronary system is patent. **(B)** Coronary angiogram shows anomalous origin of the right coronary artery (black arrowheads) from left sinus of Valsalva, next to the left (white arrowheads) coronary arteries in patient 2. Also it is noted that severe stenotic lesions are present on the RCA. **(C)** Coronary angiogram in right anterior oblique view shows collateral circulation to the distal RCA (white arrows) via septal branches of the left anterior descending artery, with the normal left coronary artery system in patient 3. (CT: computed tomography; AO: aorta; LMC: left main coronary artery; PA: pulmonary artery; RCA: right coronary artery).

**Figure 2:**
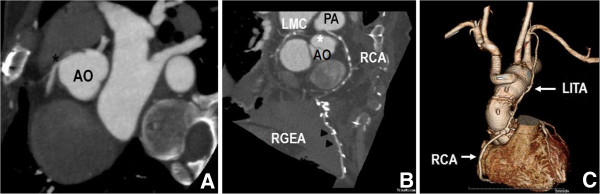
**Postoperative images. (A)** Post-operative enhanced chest CT scan shows neo-ostium (black asterisk) from the right sinus of Valsalva in patient 1. **(B)** Post-operative contrast-enhanced CT scan shows the anomalous RCA shared the same sinus with the left coronary system (white asterisk), with its attenuated proximal portion passing between the aorta and pulmonary trunk in patient 2. This image also shows that RGEA was bypassed to the distal RCA. **(C)** Post-operative three-dimensional volume rendering of contrast-enhanced CT scan in patient 3. Chest CT scan revealed that the ascending aorta and part of the aortic arch were replaced with a prosthetic graft, and that the innominate artery was bypassed using a side-arm branch. Also the left internal thoracic artery to the proximal right coronary artery bypass was patent. (CT: computed tomography; AO: aorta; LMC: left main coronary artery; PA: pulmonary artery; RCA: right coronary artery; LITA: right internal thoracic artery; RGEA: right gastroepiploic artery).

A 39-year-old woman visited out-patient department with exertional chest discomfort for 2 months. Pre-operative work-ups including cardiac enzymes, electrocardiogram, nuclear perfusion scan, and echocardiogram were carried out and the results were normal without any ischemic signs. Computed tomography (CT) revealed ARCA (Figure 1A). We thought that panic symptoms on physical exertion were caused due to ischemia and decided for a surgical correction. Under cardiopulmonary bypass and cardioplegic arrest, a neo-ostium was created on the right coronary sinus at the nearest site from the right coronary artery (RCA). After that, a right coronary arteriotomy was made on its opposite site. Anastomosis between them was performed (Figure 2A). The postoperative course was uneventful and she remains asymptomatic for 22 months.

### Patient 2>

A 61-year-old woman was emergently admitted for chest pain and diagnosed with acute myocardial infarction. Cardiac catheterization revealed that patient had triple vessel disease concomitant with ARCA (Figure 1B), and chest CT confirmed ARCA. An emergent off-pump coronary artery bypass was performed. The internal thoracic arteries (ITAs) and the right gastroepiploic artery (RGEA) were prepared. Then, the resected right ITA was anastomosed to *in situ* the left ITA in a “Y” configuration. The distal anastomoses were constructed in the following sequence: the distal left ITA to the left anterior descending artery (LAD) and the right ITA to the two obtuse marginal branches sequentially. Subsequently, the distal RCA was revascularized with the *in situ* RGEA (Figure 2B). The postoperative course was uneventful, and she remains asymptomatic for 14 months.

### Patient 3>

A 44-year-old man was emergently admitted for intractable chest pain. He had suffered from intermittent chest pain for several months. Echocardiogram revealed hypokinesia in the territory of the RCA. Subsequent cardiac catheterization revealed that there were no significant lesions on the left coronary systems, but the RCA ostium was invisible. Also it showed collateral circulation to the distal RCA via septal branches of LAD (Figure 1C). Additional aortography showed a contrast filling to false lumen via an intimal tear. Transesophageal echocardiography and chest CT confirmed acute type A aortic dissection, and he underwent emergent surgery. During the surgery, ARCA with slit-like ostium was incidentally detected. The ascending aorta and part of the aortic arch were replaced, and the RCA was bypassed by the left ITA with proximal ligation (Figure 2C). The postoperative course was uneventful, and he remains asymptomatic for 6 months.

Although difficult to estimate the exact prevalence, published data suggest that the incidence of anomalous origin of the coronary artery from opposite sinus (AOCA) may be around 0.1 ~ 0.3% up to 1.07%. ARCA is estimated to be six to ten times more common than anomalous origin of the left coronary artery (ALCA) [1,3].

Patients are usually asymptomatic. However, it could cause exertional syncope, angina, palpitations, and even sudden cardiac death (SCD) [4]. Thus, AOCA has concluded that it is a potentially dangerous anomaly and should be considered to apply aggressive treatment if patients have any ischemic signs [3,5]. It has been described that SCD in ALCA happens more common than ARCA [3]. However, we believe that physicians can encounter major adverse cardiac events happening within ARCA patients more frequently than ALCA patients since there are more absolute number of cases of ARCA than ALCA.

The diagnosing ARCA is often made incidentally, because many tests including physical exam, ECG and exercise stress testing are generally unremarkable for diagnosis of ARCA [4]. In a review of the literature by Basso and colleagues, they concluded that many tests including 12-lead ECG, maximal effort stress ECG, and myocardial perfusion scintigraphy would be unlikely to provide clinical evidence of myocardial ischemia and would be unreliable for clinical recognition of these anomalies. They also mentioned that premonitory symptoms including syncope or chest pain occurred not uncommonly in about one third shortly before sudden death [4]. In patient 1, we thought that panic symptoms on physical exertion were caused due to ischemia and decided for a surgical correction despite of negative functional tests. Fortunately, recently multi-detector CT scanners provide excellent spatial resolution allowing visualization of the coronary anatomy, all coronary artery anomalies become easy to diagnose [6]. We believe that much more AOCA cases could be detected faster and easier than ever after using a multi-detector CT scanner as a diagnosing tool. Also, magnetic resonance angiography can be used to identify anomalous vessels [3]. However, when emergency surgery is required, it is difficult to perform multi-detector CT scanner or magnetic resonance angiography in all patients because of unstable hemodynamics and the time required for the scan [7]. In our cases, multi-detector CT scanner or magnetic resonance angiography could not be performed because of an emergency situation and unstable hemodynamics in patient 2 and because ARCA was incidentally diagnosed during surgery in patient 3.

Ultimately, it is important to understand the pathophysiology of AOCA treatment to manage this condition effectively. The proposed mechanisms include slit-like ostium, acute angulation, impinged or spastic intramural proximal portion, and flattening of the interarterial segment from compression between the great vessels [2,8,9].

Multiple therapeutic options have been suggested. According to medical journals, some authors described some efficacy of medical therapy with nitrates, calcium or β-blockade, or anti-arrythmic drugs [2,10]. However, for AOCA, original problem of this condition cannot be possibly corrected by medication. Therefore, we are suggesting that this issue deserves consideration. Percutaneous coronary angioplasty has also been advocated by some physicians and reported feasible short-term results [11,12]. However, in our opinions, these reports have failed to establish a widely accepted consensus due to its rareness and no long-term results. Furthermore, this cannot effectively treat ostial issues that might be present, and leaves a long stent length that could be prone to late occlusion even with drug-eluting stent [13]. If it would show superior or equal advantages to surgical correction from long-term results, it might be a feasible strategy.

Multiple surgical options were advocated including coronary artery bypass (CABG), coronary reimplantation, pulmonary artery translocation, neo-ostium formation, and unroofing of the intramural segment [14]. Surgical strategy of choice for ARCA is quite controversial. Coronary reimplantation is one of the most physiologically beneficial procedures, but is technically difficult, and stenosis may occur at the anastomotic site. CABG is technically feasible, but the arterial conduit has a competitive flow problem if no stenotic lesions are present on the natural coronary artery. Also, a vein conduit may be problematic if the patient is young because of long-term patency. Some authors reported that favorable results were achieved with unroofing procedures [15]. However, it may cause valve insufficiency if the anomalous coronary artery is located under the valve commissure [9].

In our cases, the most interesting point is that each patient was placed in different situations.

In case of patient 2, the most interesting point is about graft selection for the RCA. The ideal bypass conduit in RCA system remains controversial, especially after bilateral ITA would be used up for the left coronary artery system. We selected RGEA because the arterial conduit may be expected to have a long-term patency compared to the vein graft because of high grade stenosis. To the best of our knowledge, this report is the first to describe in situ RGEA to RCA bypass in a case of ARCA. However, a longer follow-up period should be required to evaluate a long-term patency.

In case of patient 3, ARCA was incidentally diagnosed during surgery. If not recognized right after preoperative evaluations, it could result in serious complications perioperatively. Firstly, ARCA could be injured iatrogenically due to its abnormal course and position. So when incidentally detected in the operative field, careful dissection and suture are essential to avoid iatrogenic injury. Secondly, it could be a matter how to achieve appropriate myocardial protection. When a patient has a slit-like or flap-like opening, cardioplegia could not be delivered effectively by direct cannulation of the coronary ostium, which could result in inadequate myocardial protection. In this situation, retrograde cardioplegia delivery would be an alternative strategy. In our case, we could not deliver direct antegrade cardioplegia through the RCA ostium because of its slit-like opening and suspiciously proximal stenosis of the RCA on preoperative angiogram. We carefully dissected the aortic root and the proximal RCA. After the RCA was ligated and divided at the level of its origin, a coronary probe was passed into the RCA to confirm patency. Then antegrade cardioplegia was infused into the RCA using a small cannula.

## Conclusions

ARCA is a potentially dangerous anomaly and should treat aggressively if any ischemic signs are present and surgery is a treatment of choice for ARCA. For the better outcomes, it is important to understand anatomic and hemodynamic characteristics of each patient and appropriately select the surgical options considering each characteristic.

## Consent

Written informed consent was obtained from the patients for publication of this case report and all accompanying images. A copy of the written consent is available for review by the Editor-in-Chief of this journal.

## Abbreviations

ARCA: Anomalous origin of the right coronary artery from the left sinus of Valsalva; AOCA: Anomalous origin of the coronary artery from the opposite sinus of Valsalva; ALCA: Anomalous origin of the left coronary artery from the right sinus of Valsalva; CT: Computed tomogram; RCA: Right coronary artery; LAD: Left anterior descending artery; ITA: Internal thoracic artery; RGEA: Right gastroepiploic artery; CABG: Coronary artery bypass graft; SCD: Sudden cardiac death; ECGE: Electrocardiogram.

## Competing interests

The authors declare that they have no competing interests.

## Authors’ contributions

WH: participated in the management and wrote the manuscript. HM: performed surgery on the patient and revised the manuscript. DK: revised the manuscript. HJ: revised the manuscript. YH: revised the manuscript. HS: revised the manuscript. SK: revised the manuscript. HL: revised the manuscript. IR: revised the manuscript. All authors read and approved the final manuscript.

## Authors’ information

HM - Is a surgeon of the Thoracic and Cardiovascular Surgery department at the Haeundae Paik Hospital where the patient underwent operation. He is also an assistant professor at the Inje University College of Medicine in Busan, Korea.

## References

[B1] Angelini P Coronary artery anomalies–current clinical issues: definitions, classification, incidence, clinical relevance, and treatment guidelines Tex Heart Inst J 2002 9 4 271 278 12484611 PMC140289

[B2] Yuan SM Tager S Raanani E Anomalous origin of the right coronary artery from the left coronary sinus Chang Gung Med J 2009 9 4 455 458 19664353

[B3] Penalver JM Mosca RS Weitz D Phoon CK Anomalous aortic origin of coronary arteries from the opposite sinus: a critical appraisal of risk BMC Cardiovasc Disord 2012 http://www.biomedcentral.com/1471-2261/12/83 10.1186/1471-2261-12-83 PMC3502461 23025810

[B4] Basso C Maron BJ Corrado D Thiene G Clinical profile of congenital coronary artery anomalies with origin from the wrong aortic sinus leading to sudden death in young competitive athletes J Am Coll Cardiol 2000 9 1493 1501 10.1016/S0735-1097(00)00566-0 10807452

[B5] Taylor AJ Rogan KM Virmani R Sudden cardiac death associated with isolated congenital coronary artery anomalies J Am Coll Cardiol 1992 9 3 640 647 10.1016/0735-1097(92)90019-J 1512344

[B6] Ten Kate GJ Weustink AC Feyter PJ Coronary artery anomalies detected by msct-coronary angiography in the adult Neth Heart J 2008 9 369 375 10.1007/BF03086181 19065275 PMC2584765

[B7] Morimoto H Mukai S Obata S Hiraoka T Incidental single coronary artery in an octogenarian with acute type A aortic dissection Interact Cardiovasc Thorac Surg 2012 9 2 307 330 10.1093/icvts/ivs016 22561294 PMC3397724

[B8] Yanagawa B Alghamdi AA Chen RB Amankwaa A Verma S Coronary artery bypass graft for anomalous right coronary artery J Card Surg 2011 9 1 44 46 10.1111/j.1540-8191.2010.01116.x 21039851

[B9] Davies JE Burkhart HM Dearani JA Suri RM Phillips SD Warnes CA Sundt TM 3rd Schaff HV Surgical management of anomalous aortic origin of a coronary artery Ann Thorac Surg 2009 9 3 844 847 10.1016/j.athoracsur.2009.06.007 19699909

[B10] Kaku B Shimizu M Yoshio H Ino H Mizuno S Kanaya H Ishise S Mabuchi H Clinical features and prognosis of japanese patients with anomalous origin of the coronary artery Jpn Circ J 1996 9 10 731 741 10.1253/jcj.60.731 8933235

[B11] Rudan D Todorovic N Starcevic B Raguz M Bergovec M Percutaneous coronary intervention of an anomalous right coronary artery originating from the left coronary artery Wien Klin Wochenschr 2010 9 15–16 508 510 20676783 10.1007/s00508-010-1420-3

[B12] Hong LF Luo SH Li JJ Percutaneous coronary intervention with anomalous origin of right coronary artery: case reports and literature review J Geriatr Cardiol 2013 9 2 205 209 23888182 10.3969/j.issn.1671-5411.2013.02.011 PMC3708062

[B13] Fedoruk LM Kern JA Peeler BB Kron IL Anomalous origin of the right coronary artery: right internal thoracic artery to right coronary artery bypass is not the answer J Thorac Cardiovasc Surg 2007 9 456 460 10.1016/j.jtcvs.2006.10.011 17258583

[B14] Ho JS Strickman NE Anomalous origin of the right coronary artery from the left coronary sinus: case report and literature review Tex Heart Inst J 2002 9 37 39 11995848 PMC101267

[B15] Romp RL Herlong JR Landolfo CK Sanders SP Miller CE Ungerleider RM Jaggers J Outcome of unroofing procedure for repair of anomalous aortic origin of left or right coronary artery Ann Thorac Surg 2003 9 2 589 596 10.1016/S0003-4975(03)00436-3 12902110

